# Cellular specificity of androgen receptor, coregulators, and pioneer factors in prostate cancer

**DOI:** 10.1530/EO-22-0065

**Published:** 2022-09-08

**Authors:** Damien A Leach, Rayzel C Fernandes, Charlotte L Bevan

**Affiliations:** 1 Division of Cancer, Imperial Centre for Translational & Experimental Medicine, Imperial College London, Hammersmith Hospital Campus, London, UK

**Keywords:** androgen receptor, coregulators, pioneer factors, cell specificity

## Abstract

Androgen signalling, through the transcription factor androgen receptor (AR), is vital to all stages of prostate development and most prostate cancer progression. AR signalling controls differentiation, morphogenesis, and function of the prostate. It also drives proliferation and survival in prostate cancer cells as the tumour progresses; given this importance, it is the main therapeutic target for disseminated disease. AR is also essential in the surrounding stroma, for the embryonic development of the prostate and controlling epithelial glandular development. Stromal AR is also important in cancer initiation, regulating paracrine factors that excite cancer cell proliferation, but lower stromal AR expression correlates with shorter time to progression/worse outcomes. The profile of AR target genes is different between benign and cancerous epithelial cells, between castrate-resistant prostate cancer cells and treatment-naïve cancer cells, between metastatic and primary cancer cells, and between epithelial cells and fibroblasts. This is also true of AR DNA-binding profiles. Potentially regulating the cellular specificity of AR binding and action are pioneer factors and coregulators, which control and influence the ability of AR to bind to chromatin and regulate gene expression. The expression of these factors differs between benign and cancerous cells, as well as throughout disease progression. The expression profile is also different between fibroblast and mesenchymal cell types. The functional importance of coregulators and pioneer factors in androgen signalling makes them attractive therapeutic targets, but given the contextual expression of these factors, it is essential to understand their roles in different cancerous and cell-lineage states.

## Introduction

**Figure 1:**
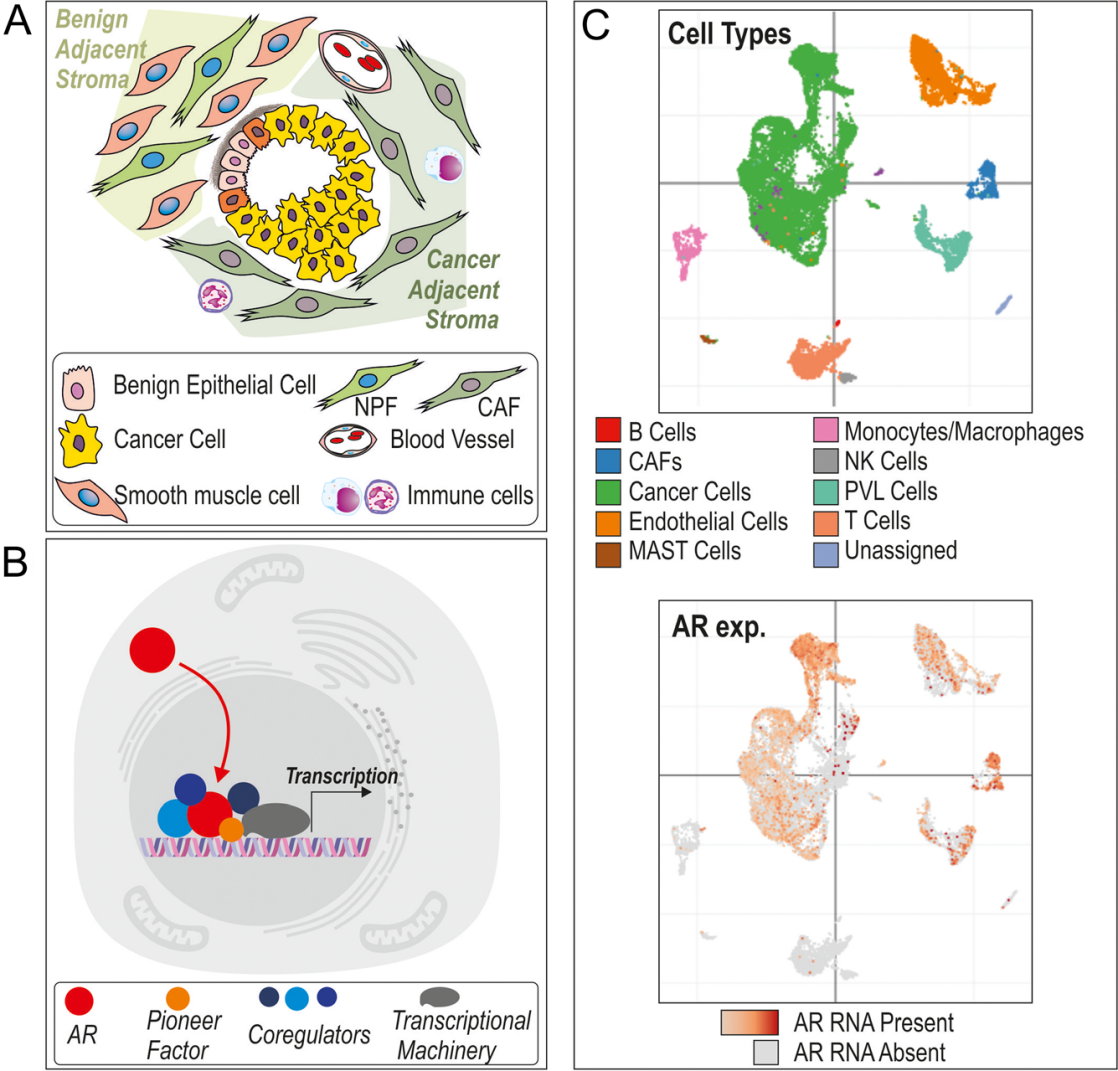
(A) Schematic of the cellular composition of the benign and cancerous prostate. In normal/benign prostate, the epithelial cells are embedded in a stroma of smooth muscle cells and fibroblasts (normal prostatic fibroblasts; NPFs), as well as other cell types such as immune and vasculature. In cancer, tumour cells develop from the benign epithelial cells, and the stroma changes to become primarily composed of cancer-associated fibroblasts (CAFs). (B) Schematic of basic AR signalling. When activated by testosterone or DHT, AR translocates from the cytoplasm to the nucleus, where it binds to chromatin, interacting with pioneer factors, coregulators, and transcriptional machinery to mediate gene expression. (C) Single-cell RNA-seq analysis of primary prostate cancer, depicting the different cell types present and the level of AR expression (shades of red) (Wu *et al.* 2021).

The prostate is an androgen-dependent organ comprised of two main compartments, which both transform with cancer progression. The normal prostate is composed of tubes of columnar epithelial cells and basal cells, which are embedded in a surrounding stromal compartment comprised of smooth muscle cells, fibroblasts, vasculature, and immune cells (Fig. 1A). Cancer develops from the epithelial compartment (Fig. 1A), where it can be preceded by an inflammatory state, prostate intraepithelial neoplasia (PIN). Uncontrolled proliferation extends tumorous growths into the glandular lumen as well as through the basement membrane into the stroma. Cancer cells are then able to invade through the stroma to local sites and metastasise to distant sites.

When the cancer metastasises, it forms new microenvironments composed of cells of the organ it has invaded. In both primary and metastatic lesions, the microenvironment is largely composed of activated fibroblasts (termed cancer-associated fibroblasts (CAFs)); immune cells are also recruited, endothelial cells are usurped to feed the tumour, and organ-specific cells are also present (Fig. 1A). There is a complex relationship between the cancer and the cells of the microenvironment, with the exchange of factors between the two influencing changes and promoting progression.

In advanced and metastatic disease, the principal treatment involves systemically targeting the action of the androgen receptor (AR) using androgen deprivation or anti-androgens. While initially effective in reducing tumour burden, the disease can re-emerge in a therapy-resistant state termed castrate-resistant prostate cancer (CRPC). This stage of the disease is less responsive to androgens/anti-androgens, but AR signalling still remains active. There are several hypotheses as to how tumours become CRPCs (reviewed extensively elsewhere, (Coutinho *et al.* 2016, Fontana & Limonta 2021)); this review will explore the roles of pioneer factors and coregulators, which are proteins that interact with AR and mediate signalling. Their ability to regulate AR activity makes them appealing therapeutic targets (Dahiya & Heemers 2022). However, it should be noted that coregulator expression changes with disease and that AR signalling is not confined to cancer cells, thus drugs targeting coregulators may have different effects with disease progression and may affect AR signalling in non-cancer tissue with unknown consequences. This review will examine the expression of AR-interacting proteins in cancer cells and the cells of the microenvironment and how their expressions and roles change with cancer progression.

## Basics of androgen receptor signalling and cellular specificity

AR regulation of gene transcription is a highly co-ordinated process (Senapati *et al.* 2020) (Fig. 1B). Upon activation by binding to testosterone, or its derivative dihydrotestosterone (DHT), the androgen receptor (AR) translocates from the cytoplasm, along microtubules, through nuclear pore complexes into the nucleus. The AR then binds to chromatin and co-ordinates with transcriptional machinery to alter gene expression. Chromatin structure and accessibility control AR binding but are actively modified by ligand activation, with increased chromatin accessibility accompanying the binding of activated AR to DNA (He *et al.* 2010, Andreu-Vieyra *et al.* 2011, He *et al.* 2012, Tewari *et al.* 2012).

**Figure 2:**
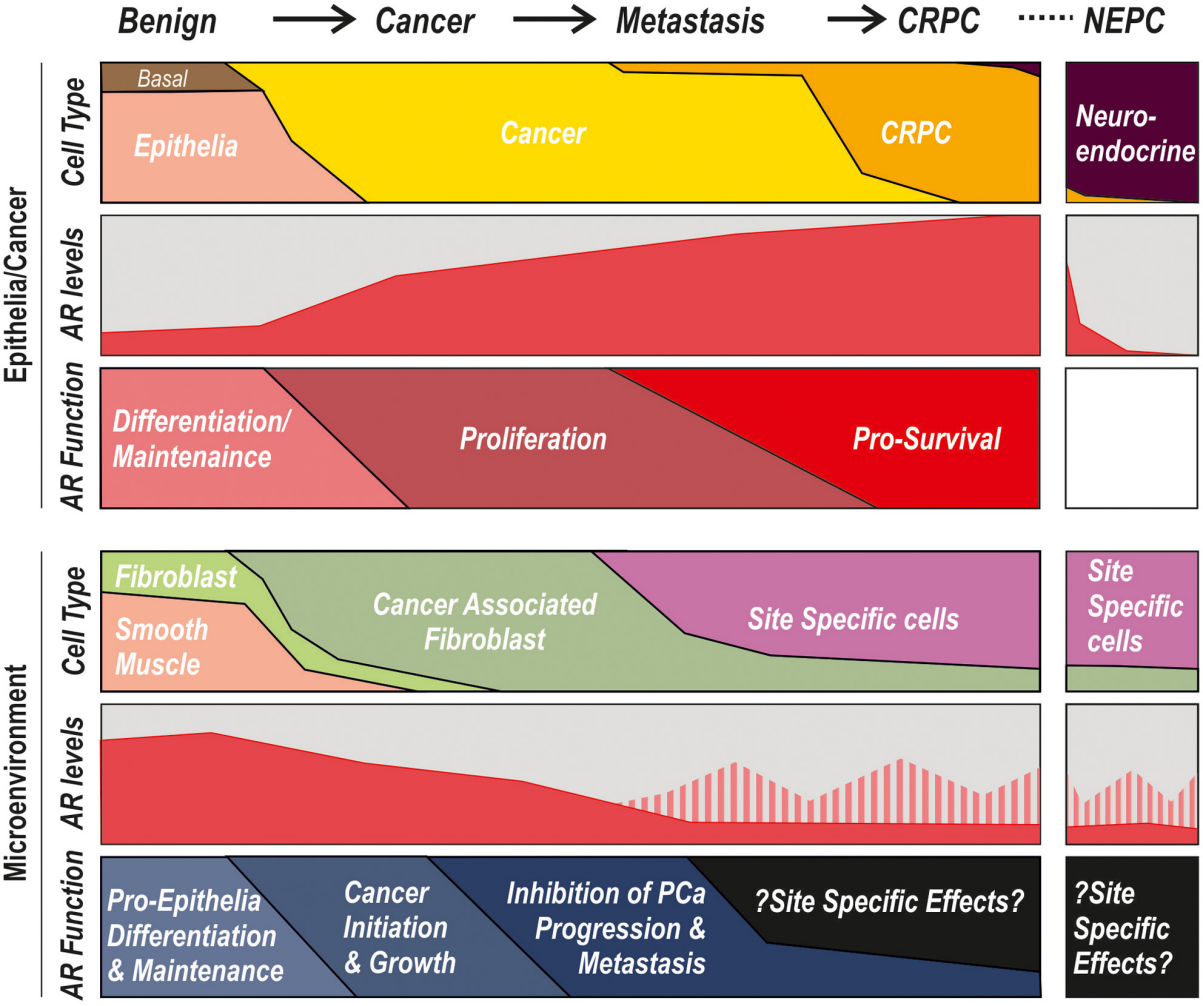
Schematic representation of AR expression and function in the epithelial/cancer compartments and stromal compartments based on the information covered in this review. In both benign and cancerous conditions, androgen receptor (AR) signalling is important for both compartments. Represented for the epithelial and stromal compartments are the cell types present, the relative levels of AR, and the main function of AR in each compartment throughout cancer progression.

AR is expressed throughout the body and heterogeneously within PCa microenvironment (Fig. 1C). AR chromatin interactions and gene regulations are both tissue- and cell-specific, with marked differences between epithelial and non-epithelial cells in terms of level of AR expression, the areas of chromatin to which AR binds, the genes and pathways regulated by androgens/AR, and the expression of AR-interacting proteins (Bebermeier *et al.* 2006, Wang *et al.* 2011, Need *et al.* 2012, Goi *et al.* 2013, Pihlajamaa *et al.* 2014) (Fig. 2). Complex patterns of cell type-specific transcription factors, nucleosome occupancy, and the acetylation and methylation of histones control cell differentiation (Teif *et al.* 2012). Indeed, specific genes are enriched with transcriptional hotspots at enhancer sites in cell type-specific patterns (Joshi 2014). This review will focus on the differences in AR signalling between epithelial cancer cells and the cells of the microenvironment.

### Expression and function of AR in epithelial/PCa cells

Recombinant mouse models have shown that epithelial AR expression is not required for embryonic prostate development (Cunha 1972, Cunha & Chung 1981, Cunha *et al.* 1987). However, epithelial AR is vital for epithelial maturation, normal epithelial function, and prostatic homeostasis (Cunha *et al.* 2004) in which it regulates genes involved in differentiation (Gao *et al.* 2001, Love *et al.* 2009).

Cancer initiation may also be driven by epithelial AR (Tomlins *et al.* 2007, Pritchard *et al.* 2009). In mouse models, AR activity, overexpression, and mutation are involved in driving changes in prostate basal and luminal epithelial cells leading to PIN and adenocarcinoma development (Stanbrough *et al.* 2001, Han *et al.* 2005*a*). During oncogenic transformation, the role of AR signalling in epithelia changes from driving differentiation to targeting genes involved in proliferation and metabolism (Gao *et al.* 2001, Xu *et al.* 2006, Wang *et al.* 2009, Massie *et al.* 2011) (Fig. 2). Meta-analysis of an AR-regulated gene signature specific to benign prostate epithelia shows that it is consistently reduced or lost in prostate cancer: the degree of loss increases with tumour grade (Stuchbery *et al.* 2016). This shift in transcriptional profile is accompanied by alterations in the patterns of AR binding to chromatin, with AR binding to regulatory regions of genes encoding drivers of proliferation that are only regulated in cancer and not in benign epithelia (Bolton *et al.* 2007, Memarzadeh *et al.* 2011, Chen *et al.* 2015, Pomerantz *et al.* 2015). Reportedly, there is only a small (16%) overlap in AR-binding sites between cancer and benign epithelial tissue (Pomerantz *et al.* 2015). Furthermore, a study evaluating AR ChIP-sequencing of patient tissue (normal *n*  = 4, localised *n*  = 4, metastatic *n*  = 3, and CRPC *n*  = 3) identified that at every stage of prostate cancer, there is a distinct binding pattern for AR (Stelloo *et al.* 2015). These differences in AR binding are potentially accompanied by distinct methylation patterns of promoter CpG islands and distinct histone markers between androgen-sensitive and insensitive disease (Wang *et al.* 2009, Mishra *et al.* 2010). Taken together, all evidence supports that the way epithelial AR interacts with chromatin, and regulates genes, alters with cancer progression.

### AR in the stromal/microenvironment

The microenvironment is composed of many different cell types, here, we will review AR in the main constituents of the cancer microenvironment; smooth muscle, fibroblast, endothelial, and immune cells.

Stromal AR is important to all aspects of prostate development and carcinogenesis (Fig. 2). Recombinant mouse models have shown that initial prostate development requires the expression of AR in the stromal smooth muscle cells, from where it influences epithelial development and glandular structure (Cunha & Chung 1981, Cunha *et al.* 2004). Similarly, during carcinogenesis, the presence of AR in stromal fibroblasts promotes the proliferation of (epithelial) cancer cells both *in vitro* and *in vivo* (Leach & Buchanan 2017). In advanced PCa, a number of studies report an inverse relationship between stromal CAF AR expression and patient outcomes: patients with no or low expression of AR in their cancer-adjacent stroma have worse outcomes compared to those with high levels of stromal AR, including cancer progression and prostate cancer-specific mortality (Mohler *et al.* 1996, Olapade-Olaopa *et al.* 1999, Ricciardelli *et al.* 2005, Li *et al.* 2008, Wikstrom *et al.* 2009, Leach *et al.* 2015, 2017*b*). In smooth muscle cells and fibroblasts, AR signalling outcomes are distinct when compared to cancer cells, only sharing between 10 and 20% overlap in genes regulated by AR/DH, and similar overlap for AR DNA-binding profiles (Berry *et al.* 2008, Tanner *et al.* 2011, Leach *et al.* 2015). Furthermore, in these fibroblast cell types, androgen both positively and negatively regulates a range of paracrine and ECM factors, such as FGFs, TGFB, WNTs, and collagens, in a cell-specific manner, which can influence cancer cells (Tanner *et al.* 2011, Lai *et al.* 2012*b*, Yu *et al.* 2013, Leach *et al.* 2015, Leach & Buchanan 2017).

Two other major cell types within the cancer microenvironment are endothelial cells and immune cells, though the role of AR within these cell types is less well understood. Both systemic endothelial cells and endothelial cells of the prostate express AR (Godoy *et al.* 2008, Annibalini *et al.* 2014), where it regulates factors that mediate angiogenesis and endothelial repair (Torres-Estay *et al.* 2015), with androgen inhibition reportedly inducing apoptosis of endothelial cells in human xenograft experiments(Godoy *et al.* 2011).

There are a variety of immune cells which express AR to varying degrees and are reported to be regulated by androgens (Mantalaris *et al.* 2001, Lai *et al.* 2012*a*, 2013) and anti-androgens (Consiglio & Gollnick 2020, Consiglio *et al.* 2020). AR appears to inhibit neutrophils and can cause neutropenia in AR-knockout male mice (Chuang *et al.* 2009), but anti-androgens are also reported to reduce excessive levels of neutrophils in females with hyper hyperinsulinaemic hyperandrogenism (Ibanez *et al.* 2005). Conversely, AR may promote the development and growth of monocytes (Consiglio & Gollnick 2020). Further to this, AR expression in macrophages has been reported to aid in prostate cancer invasion (Cioni *et al.* 2020). In B and T lymphocytes, AR regulates differentiation and cytokine production and inhibits antibody production (Olsen & Kovacs 2001, Altuwaijri *et al.* 2009).

## Pioneer factors

**Figure 3:**
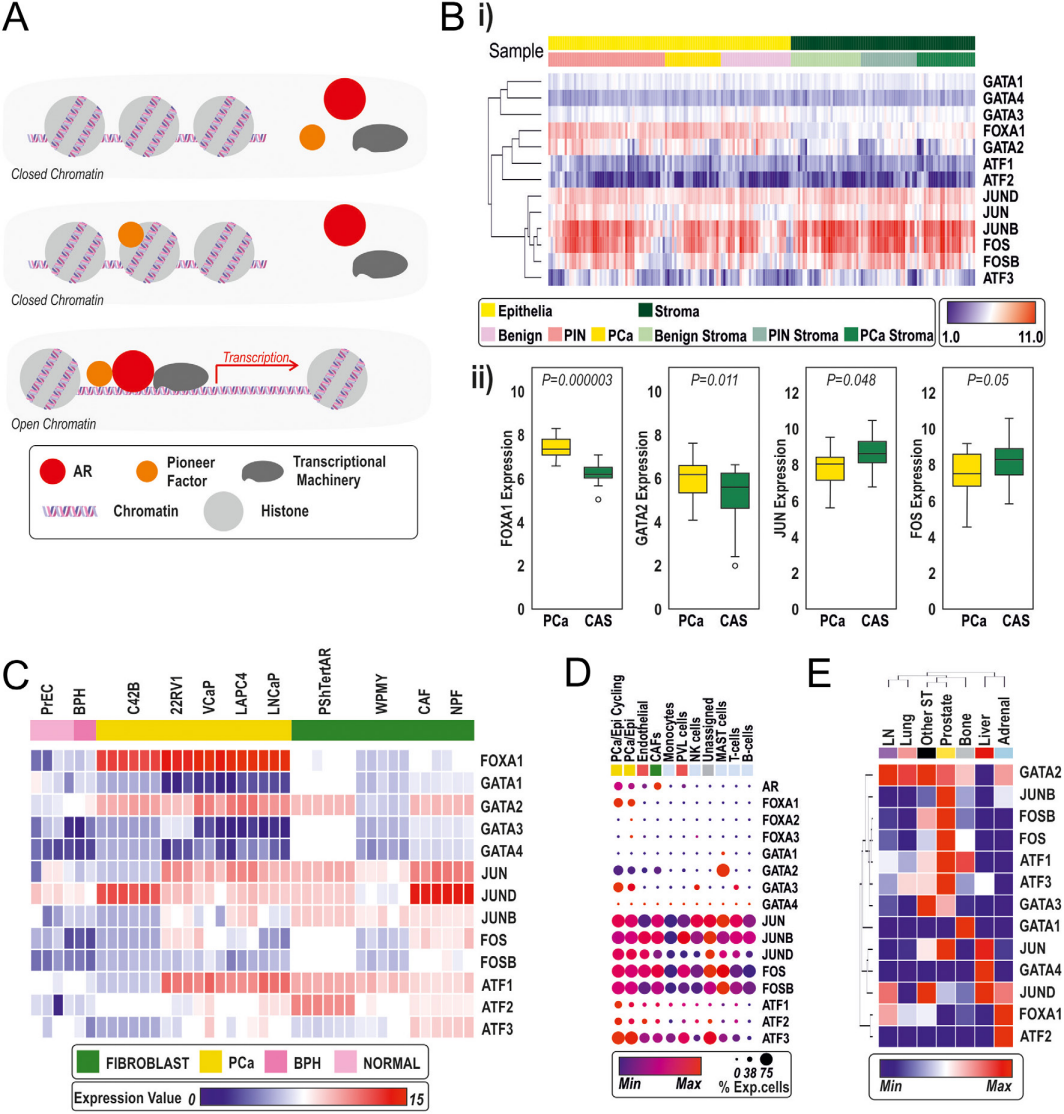
(A) Schematic of pioneer factor action, depicting binding of pioneer factors and the opening of chromatin to allow AR chromatin interactions and transcription. (B) Pioneer factor expression across cell types. (i) Samples microdissected into cancer/epithelia and stroma in benign, PIN, and cancer from patient samples. (ii) Examples of pioneer factors that are more highly expressed in cancer or stroma. (C) Analysis of pioneer factor expression in different prostatic fibroblast, benign epithelial, and cancerous epithelial cell lines. Data grouped via hierarchical clustering via Pearson correlation. Cell line data used were from GSE66850, GSE47203, GSE47354, GSE68164 (Leach *et al.* 2014, 2015, Doldi *et al.* 2015). (D) Analysis of pioneer factor expression in scRNA-seq of the primary prostate, where the size and shade of the circle represent the proportion of expressing cells and the extent of the RNA expressed. (E) Analysis of pioneer factors in metastatic sites (SU2C) (Abida *et al.* 2019). Heatmap depicts average expression of each gene for each metastatic site. Bone = 73, lymph node (LN) = 115, liver = 39, lung = 7, prostate = 7, adrenal = 1, other sites = 25. The samples investigated at each site include samples that have had either anti-androgen treatment or taxane treatment or are treatment-naïve.

One way in which AR can be regulated is through a group of proteins called pioneer factors. These are characterised as proteins which can bind to DNA regions in closed chromatin and use biochemical properties to make ‘closed’/compacted regions accessible to transcription factors and machinery (Fig. 3A). Pioneer factors interact with DNA in closed chromatin regions by passively gaining access and actively reorganising and opening chromatin (Zaret & Carroll 2011, Rodriguez-Bravo *et al.* 2017). Pioneer factors can have restricted expression across different cells and tissues, where they regulate cell-specific genes. Analysis of androgen-dependent and -independent PCa samples identified several transcription factors and pioneer factors with differential expression (Wang *et al.* 2008). Pioneer factors have the capacity to determine cell lineage (Drouin 2014) by controlling transcription factor binding at super-enhancer sites required to drive differentiation (Adam *et al.* 2015). The selective expression of pioneer factors may be the first level of control for cell-specific functions of AR (Zhang *et al.* 2010).

In benign, PIN, and PCa samples that have been microdissected into epithelial and adjacent stroma, FOXA1 is found exclusively in epithelial cells and increases in PCa compared to benign, GATA2 is predominately in epithelial cells, while AP1 components JUN and FOS are higher in stroma compared to epithelial (Fig. 3B). Cell line data suggest the same pattern of cell specificity, though there is a more marked increase in FOXA1 and GATA2 in PCa compared to benign lines (Fig. 3C). Single-cell RNA-seq data suggest that a number of other cell types, such as immune and endothelial cells, express pioneer factors similar to fibroblasts (Fig. 3D). Interestingly, when PCa has metastasised to distinct sites, the pioneer factor profile is distinct between the different metastatic sites, of which some have been exposed to a variety of anti-androgen and chemotherapy treatments (Fig. 3E).

### Pioneer factors in epithelia/PCa cells

Forkhead box protein A1 (FOXA1) is the most investigated pioneer factor in PCa as it has a prominent role in AR signalling. Before binding to chromatin, FOXA1 reads the epigenetic state of histones, such as the methylation status of histone H3 lysine 4, which then regulates the recruitment of AR to DNA (Lupien *et al.* 2008). FOXA1 has a winged-helix structure that allows it to bind to the major groove of DNA (Clark *et al.* 1993) and open chromatin for binding of AR or other transcription factors (Cirillo *et al.* 2002), and activation of AR target genes (Gao *et al.* 2003). Increased FOXA1 expression may cause increased binding of AR across the whole genome (Robinson *et al.* 2014).

FOXA1 appears to have a role in the cell specificity of AR signalling in prostate epithelial and cancer cells (Lupien *et al.* 2008, Sahu *et al.* 2013). FOXA1 and AR directly interact to regulate prostate-specific gene expression (Gao *et al.* 2003). During embryonic development, FOXA1 expression is confined to the epithelial compartment (Mirosevich *et al.* 2005) and *in vivo* models have shown that FOXA1 is required for normal prostate development and maturation as it influences the abilities of AR to regulate these processes (Gao *et al.* 2005, DeGraff *et al.* 2014). In mature prostate epithelial cells, FOXA1-KO disrupts gland structure and epithelial differentiation and alters protein marker expression (DeGraff *et al.* 2014). FOXA1 may have a role in PCa initiation, as in the mature prostate, FOXA1 is only highly expressed in the peripheral zone of the prostate, where tumours most commonly arise (Cunha *et al.* 1987, Gerhardt *et al.* 2012). In fact, on comparing the regions of AR binding between benign and cancerous epithelia, FOXA1 motifs are more highly enriched in the tumour-specific AR-binding sites (Pomerantz *et al.* 2015).

High FOXA1 expression in PCa is associated with metastasis, biochemical relapse, and overall outcome (Jain *et al.* 2011, Sahu *et al.* 2011, Gerhardt *et al.* 2012, Imamura *et al.* 2012, Robinson *et al.* 2014). The role of FOXA1 in AR signalling appears to change during cancer development and progression. In androgen-dependent PCa cell lines, FOXA1 expression or loss is unable to alter the expression response of AR target genes, PSA or TMPRSS2, to androgen, but it does affect AR binding in androgen-independent cells (Wang *et al.* 2007, 2009). FOXA1 has been suggested to be not required for AR binding to canonical AREs but is vital for AR binding to low-affinity half-site AREs that require chromatin decompaction (Jin *et al.* 2014). This may account for its association with CRPC as opposed to hormone-naïve prostate cancer.

Furthermore, FOXA1 may have an inhibitory role in EMT. Expression, ChIP, and motif analyses suggest FOXA1 is not highly involved in AR action in fibroblasts (Leach *et al.* 2017*a*) (Fig. 3B, C, D and E). Comparing PCa AR-cistrome to fibroblast AR-cistrome, the degree of overlap between the two cell types is greatly increased when FOXA1 is silenced in the epithelial prostate cancer cells (Leach *et al.* 2017*a*), which suggests a role for FOXA1 in determining phenotypically epithelial specificity of AR signalling. In support of this notion, loss of FOXA1 is reported to be vital in epithelial-mesenchymal transition (EMT) in multiple cell types (Song *et al.* 2010, Huang *et al.* 2016, Wang *et al.* 2016) including prostate cancer (Jin *et al.* 2013). In addition to inhibiting progression through EMT, FOXA1 inhibits cell motility. The mechanism behind this is still under investigation, with the possibility of an AR-independent mechanism, or through the regulation of the key EMT protein SLUG/SNAIL2 (Jin *et al.* 2013, Wang *et al.* 2016).

FOXA1 is also commonly mutated in cancer, with mutations observed in 9–11% of primary prostate cancers, up to 13% of metastatic prostate adenocarcinomas, and up to 25% of NEPC (Adams *et al.* 2019, Parolia *et al.* 2019, Beltran *et al.* 2020). A number of different missense and deletion mutations have been described, which have varying effects on AR activity (Teng *et al.* 2021). These effects of FOXA1 mutation can be achieved by either having altered affinity to chromatin, allowing for altered access to AR or by having altered interactions with the AR itself. FOXA1 mutations may also have a role in binding to AR-binding sites in the absence of AR to regulate gene expression (Baca *et al.* 2021).

GATA-binding protein 2 (GATA2) is the second most well-characterised pioneer factor in PCa and often co-localises with FOXA1 (He *et al.* 2014). GATA2 has two zinc fingers, which bind to specific nucleic acid sequences within DNA (Zheng & Blobel 2010). GATA2 has prominent role in cell lineage determination and development, mainly in haematological lines (Robert-Moreno *et al.* 2005, Lugus *et al.* 2007), but there is also an important role for GATA2 in urogenital and prostate development and in PCa (Zhou *et al.* 1998, Perez-Stable *et al.* 2000, Baca *et al.* 2021, Fernandes *et al.* 2021). GATA2 may be required for AR binding for regulatory regulation of PSA, TMPRSS2, and FKBP51, facilitating recruitment of co-activators to their promoters and subsequent upregulation of these target genes by androgen (Wang *et al.* 2007, He *et al.* 2014). GATA2 also promotes AR expression, but it is itself inhibited by AR in a negative feedback loop, so in states of low androgen (such as ADT), GATA2 levels increase, in turn increasing AR expression and activity (He *et al.* 2014).

As GATA2 expression is generally similar between normal and cancer tissue (Rodriguez-Bravo *et al.* 2017) (Fig. 3B and C), it may be more involved in advanced disease, as its expression is altered between metastatic sites, being highest in lung and lymph node metastases (Fig. 3D). In mouse models and human tissue, GATA2 expression increased with the development of CRPC (Hendriksen *et al.* 2006, Wang *et al.* 2009, Vidal *et al.* 2015), and expression levels are associated with prostate cancer aggression and outcome in patient cohorts (Bohm *et al.* 2009, He *et al.* 2014). GATA2 expression is reported to increase with ADT (Heemers *et al.* 2007), and concomitant high expression along with FOXA1 is predictive of poor clinical outcome, specifically with shorter time to biochemical relapse (He *et al.* 2014). GATA2 may have a role in metastasis, down-regulating genes involved in inhibiting invasion both *in vitro* and *in vivo* (Bohm *et al.* 2009).

GATA2 also appears to be important to AR-Vs, as well as full-length AR. The absence of GATA2 completely inhibits androgen regulation of AR-targeted genes (He *et al.* 2014). This may be due to the ability of GATA2 to facilitate co-activator recruitment needed for both AR-FL and AR-V transcriptional activity (He *et al.* 2014). FOXA1 also regulates AR-V activity in CRPC conditions; however, AR-V7-driven gene regulation is reportedly less dependent on FOXA1 than gene regulation by AR-FL (Krause *et al.* 2014, Jones *et al.* 2015).

### Pioneer factors in stroma/microenvironment

AP-1 is a transcription dimer complex composed of any combination of JUN, FOS, and ATF family proteins, although the most common arrangement is a heterodimer of c-JUN (JUN) and c-FOS (FOS). JUN and FOS act to enhance AR activity in transactivation promoter assays (Shemshedini *et al.* 1991, Bubulya *et al.* 1996, 2000, 2001, Chen *et al.* 2006). In whole/bulk tissue RNA analysis, JUN and FOS family members are differentially expressed between metastases (Chandran *et al.* 2007, Ouyang *et al.* 2008). The expression of the various AP1 component proteins in mesenchymal cells is seemingly organ-specific (Hess *et al.* 2004).

AP-1 genes are expressed in most cell types found within the prostate (Fig. 3B, C and D). As previously stated, the AR cistrome in fibroblasts is largely distinct from that of PCa cells (Leach *et al.* 2015, 2017*a*). At fibroblast-specific AR-binding sites, motif analysis highlighted that the FOXA1 motif is only marginally enriched, while AP-1 enrichment was prominent (Leach *et al.* 2017*a*, Cioni *et al.* 2018). This is mirrored by expression analysis – FOXA1 is high in cancer cells but has low expression in fibroblasts, while the proteins of AP-1 complex are more highly expressed in fibroblasts (Fig. 3B and C).

Expression of AP-1 factor JUN in the stroma regulates cancer proliferation through the secretion of paracrine factors (Szabowski *et al.* 2000, Li *et al.* 2007). These reported JUN-targeted paracrine factors are also regulated by AR (Tanner *et al.* 2011, Leach *et al.* 2017*a*) and may contribute to the ability of stromal AR to excite proliferation and development of benign and cancerous epithelia (Cunha *et al.* 2003, 2004). Interestingly, in LNCaP cells, c-JUN inhibited androgen regulation of epithelial-specific AR targets PSA and KLK2, as well as proliferation (Hsu & Hu 2013). In fibroblasts, AR inhibits proliferation but does not target PSA or KLK2, so it could be that JUN reprogrammes epithelial AR towards the profile of fibroblast transcriptome. This supports the idea that JUN is involved in stromal-specific androgen signalling.

AP-1 is also reported to be an important enhancer of EMT (Tsuji *et al.* 2008, Romagnoli *et al.* 2012, Thakur *et al.* 2014, Gao *et al.* 2015). As discussed earlier, FOXA1 appears to be inhibitory to EMT types (Song *et al.* 2010, Jin *et al.* 2013, Huang *et al.* 2016, Wang *et al.* 2016). This raises the possibility that a key process in EMT progression is a switch in the profile of pioneer factors which will then enable the expression of mesenchymal genes.

Sumoylation modulates AR in a target gene- and pathway-specific manner (Sutinen *et al.* 2014); sumoylation state is associated with AR binding to sites with distinct motifs, where AR sumoylation changes associated motifs from FOXA1 and C/EBP to AP-1 motifs (Sutinen *et al.* 2014). This suggests that sumoylation is associated with pioneer binding, and differences in the degree of sumoylation of AR between epithelial cells and fibroblasts could be involved in the cell specificity of pioneer factor interaction.

A final point of interest is that, while not widely expressed in the primary microenvironment, there is evidence that FOXA1 and GATA2 are expressed and functional in the specialised cells of organs such as bone, liver, and lung, to which prostate cancer can metastasise (Lau *et al.* 2006, Bernardo & Keri 2012, Lambrechts *et al.* 2018). While the area of pioneer protein function in the primary and metastatic microenvironments is under-researched, there are potential interesting concepts that may be inferred about how these proteins work in a cell-specific manner. Meanwhile, what effect their disruption has on cancer biology and its response to treatments are unknown.

## Coregulators

Diversity in AR binding and transcriptional profiles may also be the result of differential coregulator expression and function. Coregulators are a diverse group of molecules which include both proteins and folded RNAs that function to modulate AR signalling (Lanz *et al.* 1999, Heemers & Tindall 2007). Since the description of the first coregulator, nuclear receptor co-activator (NCoA) 1 (Onate *et al.* 1995), over 300 nuclear receptor coregulators/interacting proteins have been identified and advancing protein analysis techniques suggest that such a number is a significant underestimation (Lonard & O’Malley 2012, Stelloo *et al.* 2018). As reviewed by Heemers and Tindall (2007), coregulators can have a variety of roles within the cell aside from modulating transcription including cytoskeletal scaffolding, endocytosis, signal transduction, ubiquitination, sumoylation, and cell-cycle-associated processes (Heemers & Tindall 2007).

**Figure 4:**
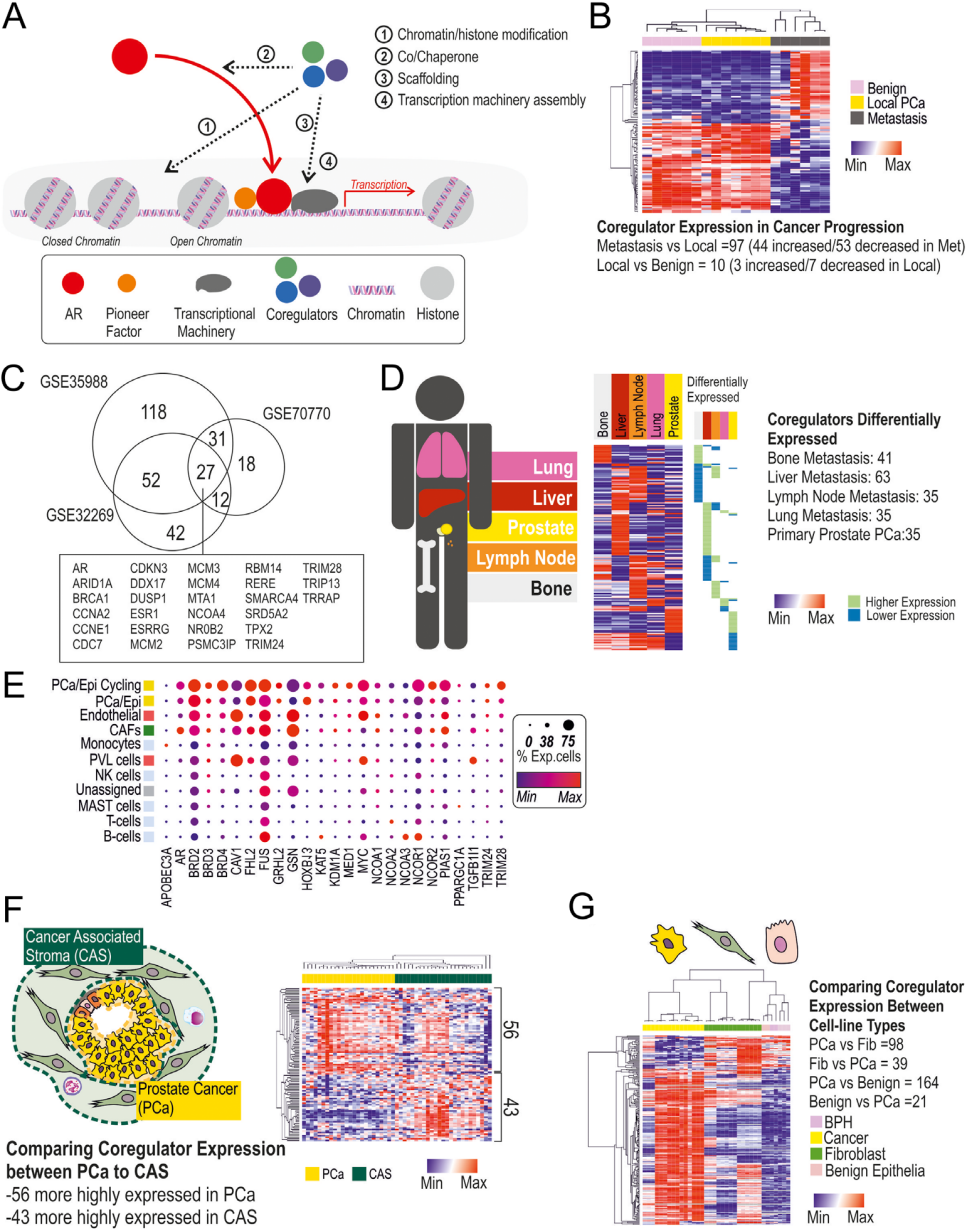
(A) Schematic of coregulators influencing AR signalling grouped into four broad categories of coregulatory action. (B) Heatmap depicting the expression of coregulator/NR-interacting proteins across benign, cancerous, and metastatic PCa samples (GSE3325). Data presented as relative to each row and grouped via hierarchical clustering via Pearson correlation. (C) Venn diagram of coregulator/NR-interacting proteins differentially expressed in CRPC samples compared to hormone-naïve cancers from three datasets (GSE35988, GSE32269, GSE70770). Differential expression set as *P* < 0.05, +/− 1LFC. The box insert identifies the genes with common differentially expression between all three cohorts. (D) Gene expression analysis of coregulators/NR-interacting proteins in metastatic sites (SU2C) (Abida *et al.* 2019). Heatmap depicts the average expression of each gene for each metastatic site. Green and blue bars indicate which gene has significantly different expression compared to the other metastatic sites measures (*P* < 0.05, +/− 2LFC; Green more highly expressed, blue lower expression). Bone = 73, lymph node (LN) = 115, liver = 39, lung = 7, prostate = 7. The samples investigated at each site include samples that have had either anti-androgen treatment or taxane treatment or are treatment-naïve. (E) Analysis of coregulator expression in scRNA- seq of the primary prostate, where the size and shade of the circle represent the proportion of expressing cells and the extent of the RNA expressed. (F) Heatmap depicting the coregulator/NR interacting proteins that are differentially expressed between different cell types in microdissected prostate cancer samples. (G) Heatmap depicting the coregulator/NR interacting and differentially expressed between different cell types; prostatic fibroblast (green), benign epithelial (pink), and cancerous epithelial (yellow) cell lines GSE66850, GSE47203, GSE47354, GSE68164 (Leach *et al.* 2014, 2015, Doldi *et al.* 2015).

Coregulators influence AR transcriptional activity in two overarching modes which can be broken down further (Fig. 4A). First, when the AR is bound to DNA, coregulators can affect chromatin structure and accessibility via remodelling of nucleosomes and/or histone modifications, thus controlling the recruitment of transcriptional machinery. Secondly, coregulators can affect the dynamic activity of the AR as a protein, influencing such parameters as stability, ligand binding, intracellular movement, and interaction with other coregulators. In the first instance, AR-associated coregulators either directly modify histones or interact with/recruit other proteins that can. Histone modifications, including methylation, acetylation, and ubiquitylation, disrupt interactions between DNA and histones, thus altering the structure of chromatin to either expose and allow gene transcription or close and inhibit transcriptional machinery from forming DNA. However, gene transcription can still be inhibited if chromatin is open. While acetylation is generally permissive to open chromatin and transcriptional activation, the effects of methylation and ubiquitylation depend on the histone residue being modified (Egger *et al.* 2004). Some coregulator proteins that catalyse histone modifications are also able to modify the AR itself, affecting its stability and consequently transcriptional activity (Coffey & Robson 2012).

Traditionally, coregulators have been seen to either enhance (co-activate) or inhibit (co-repress) the transcriptional capacity of nuclear receptors. It is now becoming apparent that coregulator action can be gene-specific, i.e. whether it acts on AR as a co-activator, a co-repressor, or has no effect, is dependent on the target gene (Purcell *et al.* 2012, Leach *et al.* 2014, Wu *et al.* 2014). Whole transcriptome analysis reports that the effect of some coregulators (presumably not direct histone modifiers) on AR-regulated transcription can be gene-specific, with silencing of individual coregulators either enhancing, inhibiting, or reversing the effect of androgen stimulation of AR (Leach *et al.* 2014, Liu *et al.* 2017). This makes the context of coregulator expression highly complicated yet incredibly important in PCa progression. Importantly, the expression of coregulators varies considerably between organs, and even within tissues (Smith & O’Malley 2004, Scarpin *et al.* 2009), with somatic expression changes implicated in a multitude of disease states (Lonard *et al.* 2007).

From benign to primary/localised PCa then to metastasis, there are changes in coregulator expression (Fig. 4B and Table 1). A suggested means by which PCa maintains AR signalling in the absence of circulating androgens in CRPC is through altered coregulator expression. In three CRPC cohorts, between 100 and 200 of the over 300 AR-interacting proteins are differentially expressed in CRPC compared to hormone-naïve PCa, with 27 differentially expressed in all 3 cohorts (Fig. 4C). Coregulator expression in PCa also alters in metastasis, with the expression profiles being, in some instances, distinct between different metastatic sites (Fig. 4D and Table 2). Single-cell RNA-seq of PCa tissue, microdissected PCa samples, and cell lines revealed distinct patterns of coregulator expression within different cell types (Fig. 4E, F, G and Table 3). Several members of the NCOA and bromodomain families were more highly expressed in the cancer cells, while CAV1, FHL2, MED1, and Hic-5 (TGFB1I1) were all higher in the fibroblasts. In the following section, we will give examples of coregulators that are specific for epithelial or stromal compartments, and changes in their expression during cancer progression that may be involved in disease advancement.

**Table 1:** Differential expression of RNA of AR-interacting proteins cancer progression.

A) AR-interacting proteins differentially expressed between metastasis and localised PCa (bulk sequencing)
FOXG1, AURKA, CDKN3, TPX2, MCM2, SRD5A1, APOBEC3B, CDC7, CDC25B, HEY1, CCNA2, PROX1, CDC25A, MCM4, SQSTM1, CCNE1, SOX4, HMGB2, BRCA1, PSMC3IP, MUC1, FEN1, CDT1, CDKN1C, E2F1, CRABP2, SPEN, MED14, H2AZ1, E2F3, SMARCA4, ITGB3BP, TRIP13, TSC2, MCM8, TBL1XR1, CTNNB1, E2F2, RPL7, MCM10, RORA, PIAS2, PRMT1, PRAME, SF3A1, FOXO1, APPBP2, NQO1, PIAS1, LMO4, AKR1C3, HSD17B11, IDE, BCL11A, HDAC4, NCOA4, NR2F2, LINC00312, ANP32A, CDK7, RORC, UBR5, GSN, SIN3A, JAZF1, LATS2, HIPK3, JUN, TP53, NR4A3, TRIP6, RORB, PRMT6, GADD45B, CITED2, CCND1, RB1, SMARCA2, SPDEF, SVIL, SRD5A2, PTEN, FLNA, DDX17, GATA3, PGR, TGFB1I1, DUSP1, CDK6, PLAGL1, FHL2, CAV1, ESRRG, ESR1, NR4A2, TCF21, TAGLN.
B) AR-interacting proteins differentially expressed between localised PCa and benign (bulk sequencing)
ESRRG, FKBP5, CRABP2, BCL11A, TBL1X, PPARGC1A, PGR, GATA3, PPARG, RORB.

**Table 2:** Expression of the genes of AR-interacting proteins in different metastatic sites compared to samples from all other sites (bulk sequencing).

Bone metastasis	Higher expression in bone metastasis
	BAG1, JAZF1, JDP2, VDR, APOBEC3A, CCND3, CMTM2, E2F2, E2F4, FOXO4, NCOA4, PADI4, PTEN, PTK2B.
	Less expression in bone metastasis
	NR2F2, PROX1, SIX3, PRPF6, NCOA5, PELP1, RERE, SREBF2, SUPT6H, ZMIZ1, ZMIZ2, KDM1A, CRTC3, EHMT2, KAT7, MED24, NCOA6, NCOR2, PARP1, PIAS4, PPARD, PPP5C, PSMC4, RAD54L2, SAFB, SMARCA4, SRCIN1, TRIM28, ZNF318.
Liver metastasis	Higher expression in liver metastasis
	PROX1, SIX3, BAG1, APPBP2, NCOA1, NUMA1, PRKCD, UNC45A, HDAC3, CDK9, DAP3, RACK1, GSN, HSPA8, LATS2, PIAS3, PRMT1, PRMT2, RCHY1, RPL7, RPL7A, SMARCA2, AKR1C1, APOBEC3B, GADD45A, HNF4A, SATB1, SULT1E1, HSD17B7, SRD5A1, FEN1, AKR1C2, AURKA, BCL11B, BRCA1, CCNE1, CDC25A, CDC25B, CDC7, E2F1, NR1H4, NR6A1, HHEX, HSD17B13, HSD17B2, JUN, NR1H3, MCM2, MCM3, MCM4, MCM8, MUC1, NR0B2, NR1I2, POU4F2, PPARA, PPARGC1A, RORA, SOX4, SQSTM1, SRD5A2, TPX2.
	Less expression in liver metastasis
	NR2F2, PROX1, SIX3, BAG1, APPBP2, NCOA1, NUMA1, PRKCD, UNC45A, HDAC3, CDK9, DAP3, RACK1, GSN, HSPA8, LATS2, PIAS3, PRMT1, PRMT2, RCHY1, RPL7, RPL7A, SMARCA2.
Lymph node metastasis	Higher expression in lymph node metastasis
	NR2F2, PRPF6, NCOA5, PELP1, RERE, SREBF2, SUPT6H, ZMIZ1, ZMIZ2, APPBP2, NCOA1, NUMA1, PRKCD, UNC45A, SF1, AR, DDX54, FKBP4, FKBP5, FLII, HDAC1, NRIP2, PDPK1, RELA, SPDEF, SREBF1, TSC2.
	Less expression in lymph node metastasis
	PROX1, JAZF1, JDP2, VDR, AKR1C1, APOBEC3B, GADD45A, HNF4A, SATB1, SULT1E1.
Lung metastasis	Higher expression in lung metastasis
	HSD17B7, SRD5A1, SMARCE1, FHL2, PIAS2, SAP30, STAT3, TMF1.
	Less expression in lung metastasis
	SF1, ARID1A, CREBBP, MED12, SIN3B.
Prostate	Higher expression in primary PCa
	AKR1C2, FHL2, AKR1C3, CAV1, CCNA1, CRABP2, ESR1, ESR2, FLNA, FOXO1, HR, HSD17B8, NR4A3, NRBF2, PLAGL1, SMAD3, SUB1, TRIP10.
	Less expression in primary PCa
	PRPF6, HDAC3, FEN1, SMARCE1, AKT1, ANP32A, NELFB, DHX9, EDF1, FUS, MTA2, PA2G4, PSMC5, PSME3, SET, SMAD4, TRIM24.

**Table 3:** Expression of the genes of AR-interacting proteins in cancer vs stroma.

A) Micro-dissected tissue: cancer vs cancer adjacent stroma (CAS)
More highly expressed in PCa
ANP32A, APPBP2, AR, ARID1A, CALR, CDK5, CDK7, CMTM2, COPS5, CTBP1, EDF1, EHMT2, FKBP4, FKBP5, FUS, GADD45G, RACK1, HNF4G, HSD17B4, HSPA8, MCM2, KMT2D, MNAT1, MPG, MTOR, NCOA4, NCOA5, NCOA6, NELFB, NFYA, NONO, NUMA1, PARK7, PDPK1, PHB2, PPID, PRKCD, RANBP9, RBM14, RBM39, RERE, RPL7, RPL7A, SCAND1, SFPQ, SMAD4, SMARCA4, SOX4, SPDEF, TBL1XR1, TGIF1, TRIM24, TRIM28, TXNRD2, UNC45A, XBP1.
More highly expressed in CAS
AKR1C3, APOBEC2, APOBEC3C, BCL11B, CAV1, CDKN1C, ESR1, FHL2, FLNA, FOXO1, GADD45A, GADD45B, GATA3, GSN, HMGB2, JAZF1, JDP2, LMO4, MGMT, NR0B1, NR1D2, NR2F1, NR2F2, NR3C1, NR3C2, NR4A3, PTEN, RARA, RARB, RARG, RBFOX2, RHEB, RORA, SKI, SMAD3, SRD5A2, SVIL, TAGLN, TCF21, TGFB1I1, YWHAH, ZFPM2, ZYX.
B) Comparison between cell line models
More highly expressed in cancer cells
AIP, ANKRD11, APPL1, AR, AURKA, BAG1, BLOC1S1, BRD8, CALR, CDC7, CDK5, CDKN1C, CDKN3, CDT1, COPS5, CRIPAK, CRTC1, CRTC2, CTBP2, DAP3, DDX5, DDX54, E2F2, E2F3, EDF1, ESRRA, FAF1, FKBP4, FUS, GADD45G, GADD45GIP1, RACK1, H2AZ1, HDAC1, HDAC2, HMGB2, HSD17B1, HSD17B4, HSD17B8, MCM2, MCM3, MED30, MMS19, MTA1, NCOA4, NCOA6, NCOR2, PA2G4, PAK6, PARK7, PARP1, PELP1, PIAS3, PIN1, PPID, PRAME, PRMT1, PRMT6, PRPF6, PSMC3IP, PSMC5, PTK2B, PTMA, PTMS, PUS3, RAN, RBM14, RBM39, RNF4, RPL7, RPL7A, RXRA, SET, SF1, SIN3B, SMARCA4, SMARCD3, SMARCE1, SNW1, SPDEF, SREBF1, SREBF2, TBL1X, TDG, TGS1, TRIM24, TRIM28, TRIP13, TXN, UBE2I, UGT2B11, UIMC1, VAV3, XBP1, XRCC6, ZMIZ2, ZNF461, ZNHIT3
More highly expressed in fibroblast cell lines
APOBEC1, APOBEC2, APOBEC3A, APOBEC3C, CAV1, CCNA1, CDK6, CMTM2, EFCAB6, FHL2, FOXG1, GATA3, GSN, HHEX, HNF4A, HSD17B13, HSD17B2, HSD3B1, JAZF1, KMT2D, MUC1, NEDD4, NQO1, NR2F1, NR5A1, PADI4, PLAGL1, PPARG, PROX1, RARB, RXRG, SENP1, SMAD3, STAT3, STS, TAGLN, TCF21, TGFB1I1, ZFPM2.
More highly expressed in cancer cells
TLE5, AIP, AKT1, ANKRD11, APOBEC3B, APPL1, AR, AURKA, BAG1, BLOC1S1, BRCA1, BRD8, CALR, CARM1, CASP8AP2, CCNA2, CCND1, CCNE1, CDC25A, CDC7, CDK5, CDK7, CDK9, CDKN1C, CDKN3, CDT1, CITED2, CRTC1, DAP3, DAXX, DDX54, DHX9, E2F1, E2F2, E2F3, EDF1, EHMT2, ELL, ESRRA, FAF1, FEN1, FKBP4, FKBP5, FLII, FOXH1, FOXO4, FUS, GADD45B, GADD45G, GADD45GIP1, GMEB1, H2AZ1, HDAC1, HDAC2, HDAC3, HDAC4, HMGB2, HNF4G, HSD17B1, HSD17B11, HSD17B4, HSD17B8, MCM10, MCM2, MCM3, MCM4, MCM5, MCRS1, MED14, MED24, MGMT, MMS19, MNAT1, MTA1, MTA2, NCOA4, NCOA6, NCOR1, NCOR2, NFYC, NRIP1, PA2G4, PARK7, PARP1, PDPK1, PELP1, PIAS2, PIAS3, PIAS4, PIN1, PPARA, PPID, PPM1D, PPRC1, PRAME, PRKCD, PRMT1, PRMT5, PRPF6, PSMC3, PSMC3IP, PSMC4, PSMC5, PSME3, PTK2B, PTMS, PUS1, PUS3, RAD54L2, RARA, RB1, RBM14, RBM39, RCC1, RNF14, RNF4, RORB, RXRA, SAFB, SAFB2, SAP30, SCAND1, SET, SF1, SFPQ, SGTA, SIN3B, SMAD4, SMARCA4, SMARCD3, SMARCE1, SNW1, SPDEF, SREBF1, SREBF2, SS18, TBL1X, TBL1XR1, TDG, TGS1, THRA, TPX2, TRIM24, TRIM25, TRIM28, TRIP11, TRIP12, TRIP13, TRIP4, TRRAP, TSC2, TXN, UBE2I, UBE3A, UIMC1, UNC45A, UXT, VDR, XBP1, XRCC6, ZMIZ1, ZMIZ2, ZNF318, ZNHIT3.
More highly expressed in benign cell lines
AKR1C1, APOBEC2, APOBEC3C, CAV1, CCNA1, CYP19A1, FHL2, GATA3, GSN, HIF1A, HNF4A, HSD17B2, NQO1, NR1D1, PLAGL1, PROX1, RARB, RXRG, TAGLN, TCF21, ZFPM2.

### Coregulators in epithelial and cancer cells

A majority of coregulator research focuses, understandably, on their actions in cancer cells. This section will investigate how coregulator expression changes within epithelial cells from prostate development through various stages of cancer progression. It will also highlight the functional role of some of these proteins in these different stages/physiologies.

#### Prostate development

As androgens play such a prominent role in prostate development, it follows that coregulators are able to influence key development processes too. When mice are deficient in steroid receptor co-activator (SRC) family protein NCoA1 expression, there is reduced androgen-driven growth/mass of the prostate gland (Xu *et al.* 1998). Although, given that stromal AR signalling may be more important for development, while epithelial AR is involved in maturation and function, it is unclear if these effects are the result of altering AR action in one compartment or both. Other members of the SRC family such as NCoA2 and NCoA3 may not be essential for AR-dependent prostate development as their expression does not alter castration-induced involution of the prostate or androgen-driven regeneration (Gehin *et al.* 2002, Chung *et al.* 2007). The co-chaperone and immunophilin FKBP52 are another examples of an AR coregulator’s importance in development, with prostate dysgenesis observed in knockout mice (Cheung-Flynn *et al.* 2005).

#### Prostate cancer development/progression

The balance between AR and co-regulators/co-repressors changes with prostate carcinogenesis, metastasis, and resistance to treatment (Buchanan *et al.* 2011). Indeed, it appears that coregulators are differentially expressed throughout cancer development and progression, and such alterations are likely essential for cancer initiation (Kinoshita *et al.* 2005, Chmelar *et al.* 2007, Heemers & Tindall 2007, Heemers *et al.* 2010, Qin *et al.* 2014). Analysis of benign/normal and cancer cell lines identifies a strong pattern of differentially expressed coregulators, allowing researchers to model changes (Fig. 4G).

Studies in murine models support the hypothesis that SRC/NCoA co-activator proteins are involved in prostate cancer initiation and progression. Overexpression of NCoA2 in mouse epithelia is able to mimic the early stages of PCa (Qin *et al.* 2014). Combined with partial loss of PTEN, NCoA2 overexpression can promote invasive adenocarcinoma and metastasis (Qin *et al.* 2014). Expression of NCoA3 is similarly important for prostate carcinogenesis in transgenic mice. NCoA expression is induced in the early stages of tumorigenesis, with NCoA3 loss preventing progression and inhibiting metastasis (Chung *et al.* 2007). Targeting NCOA3 via genistein reduces cell proliferation and suppresses the development of prostate cancer in transgenic rat models (Harper *et al.* 2009), although knockout of NCOA1 does not significantly alter prostate cancer initiation and progression (Xu & Li 2003). Conversely, co-repressor protein expression/function is frequently downregulated in cancer. Nuclear repressor co-repressor 1 (NCOR1) function, for example, declines with PCa progression; expression levels in prostate tumours are reduced compared to normal tissue and are further reduced in metastasis (Pedraza *et al.* 2000).

Interestingly, coregulator expression profiles in PCa metastases are potentially dependent on metastatic site (Fig. 4E). There are reports suggesting that the cells of the microenvironment may play a role in this. In bone, PCa cells are influenced by the microenvironment and their coregulator expression and interactions are altered, creating unique AR-coregulator complexes (Berry *et al.* 2008). LNCaP cells grown in the presence of bone osteoblasts had increased SRC1/TIF2 expression and decreased SMRT/NCoR expression (Villagran *et al.* 2015). LNCaP and C4-2B cells have microenvironment-dependent AR interactions with SRC1/TIF2 and expression of NCOR/SMRT in a ligand-dependent manner (Jiang *et al.* 2000). The differing coregulator profiles may alter cancer response to anti-androgen therapy; indeed, it is noted that PCa cells in bone, liver, and lung have different responses to therapy (Shore *et al.* 2016). In animal studies where the same prostate cancer cells are injected into the prostate or bone, the subsequent response of the tumours to castration was site-dependent (Bergstrom *et al.* 2016). Taken together, these suggest that changes in the coregulatory profile within metastatic tumours may change the responsiveness of cancer cells.

#### Coregulators in CRPC

It has been proposed that alterations in coregulator levels and functions contribute to the development of CRPC (Chen *et al.* 2004). A number of coregulator/co-activators are more highly expressed in CRPC (Li *et al.* 2000, Marvin *et al.* 2000, Gregory *et al.* 2001), making targeting coregulators an attractive potential therapeutic option (Biron & Bedard 2016).

In CRPC, the SRC family has received the most attention, as three of its members with HAT activity, NCOA1, NCOA2, NCOA3, stimulate AR activity by interacting with the AF1 and AF2 regions (Bevan *et al.* 1999) and are all highly expressed in CRPC (Evans *et al.* 2000, Gregory *et al.* 2001, Taylor *et al.* 2010, Qin *et al.* 2014). NCOA2 depletion actually prevents the development of CRPC in mice models (Qin *et al.* 2014). In fact, NCOA2 is able to reprogramme AR signalling in cancer cells towards metabolism for survival from therapy (Dasgupta *et al.* 2015), while NCOA3 drives proliferation and promotes survival pathways to combat the activation of apoptosis (Hirase *et al.* 2000). *In vitro* studies investigating flutamide (another therapeutic anti-androgen) resistance identified coregulators included in the gene expression changes associated with insensitivity to flutamide, with NCOA2 and NCOA4 increasing and co-repressor NCOR1 decreasing (Straus *et al.* 2000). High NCOA3 protects prostate cancer cells from treatment with another anti-androgen, bicalutamide (Feng *et al.* 2009). These expression changes of the NCOA proteins allow for maintained AR activity in the presence of low or absent androgens in androgen-dependent and -independent cell lines (Gregory *et al.* 2001). Increases in NCOA expression also allow for AR activation by other ligands (Berger *et al.* 2000).

In recent years, coregulators containing bromodomains have been proposed as therapeutic targets in CRPC (Urbanucci *et al.* 2017, Urbanucci & Mills 2018). Bromodomain proteins BRD2,3,4 interact with and promote AR chromatin interactions and transcription of AR targets, and their inhibition can repress cancer cell proliferation (Urbanucci & Mills 2018, Faivre *et al.* 2020). Another such family, the bromodomain containing TRIM proteins (TRIM24, TRIM28), can bind to acetylated enhancer sites and with AR and have been reported as being increased with PCa progression to CRPC (Chattopadhyay *et al.* 2000, Groner *et al.* 2016).

A number of other coregulators outside of the above families are upregulated in CRPC (Fig. 4C). High expression of the prostate-specific FHL2 has been associated with recurrence and CRPC (McGrath *et al.* 2013). SWI/SNF family members, such as SMARCA4, are upregulated with disease progression and development of CRPC (Cyrta *et al.* 2020). SMARCA4 is able to modify chromatin accessibility for AR (Launonen *et al.* 2021). HOXB13 acetylates histones and its expression increases with CRPC and correlates with AR-V7 expression (Sharp *et al.* 2019), which it interacts with chromatin (Chen *et al.* 2018). FUS is an AR-interacting protein with contextual AR co-activator/co-repressor activity that has correlations with patient survival (Yoon *et al.* 2000, Brooke *et al.* 2011). GRHL2 has been shown to interact with, and also regulate, AR-Vs and may have roles in controlling phenotypic and invasive properties (Paltoglou *et al.* 2017).

Co-repressor expression and activity are usually lost by either deletion or mutation in CRPC. NCOR1 and SMRT repress AR-targeting genes and inhibit co-activator function by competitive interaction (Marra *et al.* 2000, Pedraza *et al.* 2000); their expression is lost/reduced in CRPC xenograft models (Wu *et al.* 2000). The role of NCOR1 would appear to be specific to the epithelia, as there is very little to no expression in the stroma under any conditions. Prohibitin (PHB) recruits HDAC proteins and inhibits AR activity in reporter assays through competition with co-activators (Gamble *et al.* 2007); *in vivo* it inhibits histone acetylation and prostate tumour growth (Dart *et al.* 2012). PHB is expressed in androgen-dependent PCa cells but not in CRPC cells (Nisoli *et al.* 2000).

From these data, it becomes apparent that the ratios of different co-activators and co-repressors are a vital aspect of CRPC, capable of influencing the progression of the disease. It would also appear that differential expression of coregulators alters the AR transcriptional profile in a cell-specific manner and may actually influence the phenotype of the cell, making them more or less responsive to therapy.

### Coregulators in stromal cells of the microenvironment

As AR action in non-cancer cells is a developing area of research, the role of coregulators in cells of the microenvironment such as smooth muscle cells, fibroblasts, other mesenchymal cells, and immune cells needs to be understood. Single-cell analysis shows that coregulators are widely expressed throughout all the cells of the microenvironment, with some common and others specific to the cell type (Fig. 4E). A majority of this section will focus on coregulators in fibroblasts but will also highlight emerging roles in other cell types.

#### Stromal cells prostate cancer development

As with pioneer factors, there is apparent cell specificity for coregulator expression within the prostate and ratios of coregulators can be vastly different between cancerous epithelial cells and fibroblasts (Mestayer *et al.* 2003, Bebermeier *et al.* 2006). Further, expression can be different between fibroblasts from different tissue/organ origins (Bebermeier *et al.* 2006). The ability of coregulators to alter NR activity is tissue-/cell type-dependent, for example, PR is dependent on SRC3 in breast and SRC1 in uterine tissue, but SRC3 expression has no effect on PR in uterine and SRC1 has no effect on PR in breast (Han *et al.* 2006). This insinuates that coregulator activity is cell- and site-specific (Han *et al.* 2005*b*).

Interestingly, on analysis of publicly available data sets, we found little significant difference in coregulator expression between normal prostatic fibroblasts (NPFs) and CAFs (Fig. 4G). There has been a suggestion that SRC family proteins may have altered expression in CAFs of breast cancer compared to NPFs (Tzelepi *et al.* 2009). The membrane protein CAV- 1 is a coregulator that interacts with the NTD and LBD of AR, apparently involved in N/C interaction (Lu *et al.* 2001). CAV-1 expression is noted to be increased in prostatic CAFs (Orr *et al.* 2012). Two studies of 298 and 724 PCa patients, respectively, reported a relationship between stromal CAV-1 levels and Gleason scores, disease relapse, and survival (Ayala *et al.* 2013, Hammarsten *et al.* 2016). The expression of p300 in fibroblasts increases in response to TGFB treatment and transformation to CAF phenotype (Ghosh & Varga 2007). Another coregulator, MED1, was significantly more highly expressed in fibroblasts than epithelial cells, and reports suggest that MED1 in fibroblasts can actually regulate the expression of AR target genes in a pattern distinct from other cell types (Pope & Bresnick 2013).

Interestingly, in fibroblasts, there appears to be a relationship between proteins involved in cell movement and AR. Filamin A (FLNA) is a protein involved in linking the cytoskeleton and the plasma membrane and is most highly expressed in the stroma. The FLNA–AR axis mediates CAF movement, which in turn influences cancer invasiveness (Di Donato *et al.* 2021). Hic-5 (TGFB1I1, ARA55) is a mesenchymal focal adhesion protein with a coregulator role (Heitzer & DeFranco 2006, 2007). Its expression is not noted to change in cancer progression (Wikstrom *et al.* 2009) but it does regulate AR activity as both a co-activator and co-repressor and affect fibroblast adhesion and motility (Leach *et al.* 2014). One study reported Hic-5 to become expressed in prostate cancer epithelial cells after ADT therapy (Li *et al.* 2011), and Hic-5 is also expressed in AR null-PC3 cells (in which exogenous AR signalling resembles fibroblast AR signalling) (Litvinov *et al.* 2006, Kim *et al.* 2015, Leach *et al.* 2015); perhaps, as an attempt by the cancer cells to become less epithelial, or more mesenchymal, and escape the effects of ADT treatment.

#### Stromal cells in metastasis and CRPC

In some aspects, the metastatic microenvironment is composed of similar cell types as the primary microenvironment, in that there are fibroblasts, endothelial cells, and immune cells. Whether AR action is distinct in these cell types based on their resident organ is unclear. Furthermore, specialised cells of the metastasised organs can express AR, but the role of coregulators in these cell types is largely unknown.

In the bone microenvironment, there are AR-positive osteoblasts (McCarthy & Centrella 2015, Villagran *et al.* 2015), where androgens regulate bone reabsorption and mass (Kawano *et al.* 2003). Here, AR also regulates TGFB secretion, which may have bearing on how hospitable cancer cells find the bone. The action of AR in osteoblasts is reported to be mediated by the co-repressor TOB1 (Kawate *et al.* 2005). Other coregulators such as NCOA1 and JUN are expressed in osteoblasts, but their function in terms of AR in these cells is unknown (Lau *et al.* 2006).

In the lung microenvironment, a number of distinct stromal cell types have been found that express AR and also coregulators such as FOXO3, FOS, FOXP1, and MYC (Lambrechts *et al.* 2018). The function of AR coregulators in the microenvironment cells of other organs still needs to be fully elucidated.

## Concluding remarks

As interest grows in targeting coregulators to disrupt AR signalling in CRPC and combat advanced disease, it will be important to assess the expression of potential AR coregulator targets in all the cell types present within the microenvironment at primary and metastatic sites. Additionally, as our knowledge of AR in the cells of the microenvironment increases, targeting specific coregulators may also provide novel means of treating advanced prostate cancer.

## Declaration of interest

The authors declare that there is no conflict of interest that could be perceived as prejudicing the impartiality of this review.

## Funding

The authors acknowledge funding from the Prostate Cancer Foundationhttp://dx.doi.org/10.13039/100000892 (PCF) and NIHR Imperial Biomedical Research Centrehttp://dx.doi.org/10.13039/501100013342-funding.
